# Trends in Complications and Outcomes in Patients Aged 65 Years and Younger Undergoing Total Knee Arthroplasty: Data From the American Joint Replacement Registry

**DOI:** 10.5435/JAAOSGlobal-D-22-00116

**Published:** 2022-06-15

**Authors:** Akash Shah, David Cieremans, James Slover, Ran Schwarzkopf, Morteza Meftah

**Affiliations:** New York University Langone Orthopedic Hospital, New York, NY.

## Abstract

The purpose of this study was to identify common complications and the rates of readmission and revision in total knee arthroplasty (TKA) patients younger than 65 years.

**Methods:**

Using the American Joint Replacement Registry, we conducted a retrospective review of all TKAs done in patients aged 18 to 65 years from 2012 to 2020. Demographic factors such as age, sex, Charlson Comorbidity Index (CCI), and preoperative Veterans RAND 12-Item Health Survey Physical Component Summary (VR-12 PCS) scores were collected. We excluded patients older than 65 years and revision, oncologic, and nonelective cases. Primary outcomes included cumulative revision rate, 90-day readmission rate, and reason for revision. Univariate analysis and the Kaplan-Meier method were used.

**Results:**

Six thousand one hundred seventy-nine patients were included. The average age was 58.8 years (SD 5.5 years), 61% were female, 82% were White, and 88% had a CCI of 0 (1 = 8% and ≥2 = 4%). The mean follow-up was 42.51 months. Seventy-four patients (1.2%) underwent revision. Sixty-nine patients (1.1%) were readmitted within 90 days. No factors assessed increased revision rates. Revision-free survivorship was 98.7% (95% CI 98.4 to 99.0) and 98.6% (95% CI 98.2 to 99.0) at 5 and 8 years, respectively. Infection (15%), aseptic loosening (14%), and instability (12%) were the most common indications for revision.

**Conclusion:**

TKA done in young and presumed active patients has excellent survivorship. Long-term follow-up is needed to assess survival trends in this growing population.

The demand for total knee arthroplasty (TKA) in the United States is anticipated to increase 673% from 2005 to 2030, with 5.28 million TKAs to be done annually.^1^ As average life expectancy increases, younger and more active patients are undergoing TKAs. Long-term outcomes have shown increased failure rates due to mechanical failure, polyethylene wear, instability, and infection in this patient population.^2,3^ However, these long-term studies fail to account for improvements in implant design and surgical techniques largely used today.

Several authors reported good survivorship of TKA in young and active patients at 30 years.^4,5^ The generalizability of these studies is limited because of data being supplied from only few institutions. Currently, no study has analyzed the American Joint Replacement Registry (AJRR) database for outcomes of young and presumed active patients who have undergone primary TKA. This registry provides robust data on hip and knee arthroplasties done in the United States and offers trends in outcome measures. By collecting procedural, postoperative, and patient-reported outcome measures, the AJRR database provides a standardized metric to analyze surgical trends and patient outcomes across the country.

This study analyzed the AJRR database for patients younger than 65 years who underwent TKA. The objective was to investigate the overall survivorship of these patients with revision for any reason as the end point.

## Methods

An application was submitted to AJRR in 2020 to analyze all TKAs done in patients aged 18 to 65 years from 2012 to 2020 and had documented preoperative VR-12 PCS scores. The main purpose of this study was to determine whether improved preoperative patient factors were associated with improved outcomes. Therefore, only patients who had documentation of all the above variables were included in the analysis. The AJRR collects knee and hip arthroplasty data, including procedural, postoperative, and patient-reported outcome measures, and contains 1,897,050 procedures from hospitals, ambulatory surgery centers, and private practice groups in the United States. Collected patient demographic factors were age, sex, Charlson Comorbidity Index (CCI), and preoperative Veterans RAND 12-Item Health Survey Physical Component Summary (VR-12 PCS) scores. The VR-12 PCS is derived from a patient-reported questionnaire that measures physical function, mental health, pain, energy, social interaction, and overall health.^6^ The Charlson Comorbidity Index is a method for evaluating comorbid conditions that might affect mortality and assigns an objective score to a patient.^7^

Age was stratified into four groups: 18 to 34 years, 35 to 44 years, 45 to 54 years, and 55 to 65 years. Sex was either male or female, and race was Black, White, or other. CCI was stratified into three groups: zero, 1, or ≥2, with each integer representing the number of medical conditions associated with morbidity.

Exclusion criteria included patients older than 65 years and revision and oncologic TKA cases. International Classification of Diseases 10th Revision (ICD-10) codes were used to stratify the type of case and reason for revision and readmission. Primary outcome measures included cumulative revision rate, 90-day readmission rate, and reason for revision.

Logistic regression analysis was used to evaluate the association between a preoperative VR-12 PCS and a subsequent revision or 90-day readmission adjusting for potential confounders. Statistical significance was set at *P* < 0.05. The Kaplan-Meier method was used to measure survivorship with revision for any reason as the end point.

## Results

Of the 6,179 patients included in the analysis, 61% were female, 39% were male, and they had an average age of 58.8 ± 5.5 years (Table 1). The mean follow-up was 42.51 months. Seventy-four patients (1.2%) underwent revision. Sixty-nine patients (1.1%) were readmitted within 90 days (Table 2). Most common causes for revision were infection (14.9%), aseptic loosening (13.5%), and instability (12.2%). Most common reasons for 90-day readmission without revision were infection (34.8%), pain (8.7%), and extensor mechanism disruption (4.4%) (Table 2).

**Table 1:** Demographic Summary

Factors	TKA (N = 6,179)	
	N	%
Mean age (SD)	58.8 (5.5)	
Age category		
18-34	24	0.4
35-44	89	1.4
45-54	1,049	17.0
55-65	5,017	81.2
Sex		
Male	2,411	39.1
Female	3,755	60.9
Race		
White	5,046	81.7
Black	300	4.9
Other	833	13.5
Charlson comorbidity index		
0	5,440	88.
1	475	7.7
≥2	264	4.3

TKA = total knee arthroplasty

**Table 2:** TKA Outcomes and Top Diagnoses

Revision and Readmission Rates	TKA (N = 6,179)	
Linked revisions	74	1.2%
Linked 90-d readmissions (without revision)	69	1.1%

Top 3 Reasons for Revision	TKA (N = 74)	
Infection	11	14.9%
Aseptic loosening	10	13.5%
Instability	9	12.2%

Top 3 Reasons for 90-d Readmission w/o Revision	TKA (N = 69)	
Infection	24	34.8%
Pain	6	8.7%
Extensor mechanism disruption	3	4.4%

TKA = total knee arthroplasty

**Figure 1:**
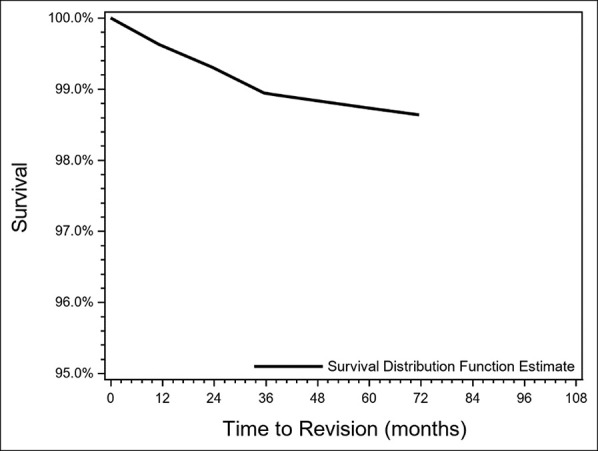
Graph showing Kaplan-Meier survivorship for total knee arthroplasty in patients yopunger than 65 years.

Readmission within 90 days requiring revision was more common in Black patients compared with White patients, although this did not reach statistical significance (*P* = 0.077) (Table 3). Age, sex, CCI, and preoperative VR-12 scores were not associated with increased early readmission requiring revision. Using the Kaplan-Meier method, patient survivorship free of revision was 99% (95% CI 99.06 to 99.49) at 2 years, 99% (95% CI 98.37 to 99.02) at 5 years, and 99% (95% CI 98.20 to 98.97) at 8 years (Figure 1).

**Table 3:** Association Between Independent Factors and Linked Revisions Among Younger Patients (18 to 65 Years)

Factors	TKA (N = 6,142)			
	Odds Ratio	Lower Limit	Upper Limit	*P*
Preoperative VR-12 PCS	0.981	0.954	1.008	0.159
Age	0.977	0.94	1.015	0.229
Sex: female versus male	1.212	0.744	1.973	0.44
Race: Black versus White	2.074	0.924	4.656	0.077
Race: other versus White	0.837	0.397	1.767	0.641
Charlson comorbidity index	0.851	0.555	1.305	0.459

TKA = total knee arthroplasty, PCS = Physical Component Summary Scores

Readmission within 90 days not requiring revision was not associated with any variables analyzed in the database (Table 4). We did not find any correlation between age and VR-12 scores with readmissions.

**Table 4:** Association Between Independent Factors and Linked 90-day Readmissions Without Revision Among Younger Patients (18 to 65 Years)

Factors	TKA (N = 53)			
	Odds Ratio	Lower Limit	Upper Limit	*P*
Preoperative VR-12 PCS	0.913	0.785	1.062	0.238
Age	1.103	0.897	1.356	0.351

TKA = total knee arthroplasty, PCS = Physical Component Summary Scores

## Discussion

With an increase in demand and broader indications for surgery, patients aged 65 years and younger are likely receiving TKAs more frequently than at any other time. Two nationwide database studies and a systematic review have evaluated the complication rates of TKA in this patient population and demonstrated similarly excellent survival rates in this patient population.^8–10^ However, these studies may not accurately reflect the advances made in implant design over the past several decades. In our analysis of the AJRR database, patient survivorship free of revision was 99% at 8 years with a 1.2% cumulative revision rate. Owing to inherent limitations of the database, these findings represent trends in data and cannot yet be used to make definitive conclusions on outcomes.

In a multicenter study by Parvizi et al,^11^ residual symptoms and functional deficits in 661 young patients with a mean age of 54 years were assessed at 1 to 4 years after primary TKA. They reported that 89% of the patients were satisfied with their capacity to do daily tasks and 91% of the patients were satisfied by the reduction in pain. Approximately 33% of these patients noted residual symptoms, including some degree of pain (33%), stiffness (41%), grinding noises (33%), and swelling and tightness (33%).^11^ Their analysis suggests that this patient population should carefully consider TKA because of the high likelihood of residual symptoms.

In a cohort of 44 TKAs, Meftah et al^12^ showed that the long-term follow-up of all-polyethylene tibial implants was 12.4 years (range 10 to 18 years) in patients aged 60 years or younger. Kaplan-Meier survivorship at 10 years was 99.7% for revision because of mechanical reasons and 95.5% for all failures.^12^ In another study by the same authors, patients aged 60 years and younger who underwent TKA with a posterior-stabilized implant design had a 99% survivorship at 12.3 years.^13^ The results of the present study show similar survivorship of TKA in young patients to the existing literature.

A study by Della Valle et al showed a 92% survivorship free of aseptic revision in 298 TKAs in patients younger than 50 years.^14^ In addition, the most common reasons for revision were aseptic loosening (4.8%), deep infection (3.2%), and polyethylene wear (2.8%).^14^ The authors noted good implant durability, with increased revision rates if the tibial cut was in excessive varus (>4^o^) or slope (>10^o^). Their analysis provided encouraging data that TKA in young patients has excellent mid-term implant survivorship. In the AJRR database, 0.2% of all TKAs (11/6179) done in patients aged 65 years and younger required revision because of infection. Comparatively, the AJRR database showed improved infection rates in this age group than in previous studies. Moreover, 90-day readmission not requiring revision in this age group was 0.4% (24/6179). Improvements in current infection mitigation during the perioperative period may support these lower infection rates.

In our AJRR database analysis, there was a trend toward higher revision rates in Black patients compared with White patients (*P* = 0.077). Lavernia et al investigated pain, well-being, and function before and after TKA using self-administered questionnaires.^15^ The authors concluded that no preoperative differences existed between races but that Black patients had worse postoperative outcomes on the Quality of Well-Being questionnaire and 36-Item Short Form Health Survey Physical Component Summary (SF-36 PCS). Furthermore, Singh et al^16^ noted that Black patients younger than 65 years were up to 5 times more likely to be discharged to an inpatient rehabilitation facility when compared with their White counterparts. Moreover, young Black patients were more likely to have a readmission within 90 days than young White patients.^16^ A growing body of the literature, including the results of this study, has shown notable racial disparities in TKA patients. Additional studies should aim to identify quality improvement measures in minority orthopaedic populations.

Our study had several limitations. First, most of the patients had a CCI of zero. As a result, this study was not designed to determine statistical significance based on comorbidity. However, given the paucity of TKAs in young and presumed active patients, we think our study is the first to analyze the largest sample size in a cohort that is generally healthier than the elderly TKA population. Second, there was no standardization of the International Classification of Diseases 10th revision diagnosis codes for revision cases or readmissions entered into the database. As a result, diagnoses for a small percentage of cases could not be determined because of the ambiguity of the International Classification of Diseases code. Those that could not be identified are listed in Appendix Table 1, http://links.lww.com/JG9/A224. Third, additional surgeries not done at AJRR sites and patients lost to follow-up were not included in the database analysis. This is an inherent limitation of all registry studies based on institutional participation as compared with national registries. As a result, the revision rates may actually be higher than that reported in the AJRR data. Fourth, the data set does not capture whether patients choose different surgeons if they required a revision surgery. As a result, failure and infection rates may actually be higher than that reported in the AJRR data. Fifth, we limited to all-time revision in an effort to maximize outcome capture because AJRR cannot account for loss to follow-up in the younger-than-65-year-old population. Sixth, collection of data at irregular intervals and inconsistent data entry could have affected the Kaplan-Meier analyses. Finally, we were not able to account for patients lost to follow-up in the target population of those younger than 65 years. Despite these limitations, we think that most of the revisions and readmissions were captured correctly and provide the largest overview to date of this population.

## Conclusion

TKA done in young and presumed active patients less than 65 years have excellent survivorship. Although racial disparities may exist with Black patients having higher revision rates than White patients, this difference did not reach statistical significance. Data from the AJRR show promising trends in TKA survivorship in this rapidly expanding patient population; however, long-term outcome studies are needed to confirm the validity of these findings and future utility of this comprehensive database.
